# An Evolutionary Game Model for Industrial Pollution Management under Two Punishment Mechanisms

**DOI:** 10.3390/ijerph16152775

**Published:** 2019-08-03

**Authors:** Chuansheng Wang, Fulei Shi

**Affiliations:** School of Management and Engineering, Capital University of Economics and Business, Beijing 100070, China

**Keywords:** evolutionary games, industrial pollution, static punishment mechanism, dynamic punishment mechanism

## Abstract

In recent years, with the rapid development of the economy, industrial pollution problems have become more and more serious. This paper constructs an evolutionary game model for industrial pollution between the local governments and enterprises to study the dynamic evolution path of a game system and the evolutionary stable strategy under two punishment mechanisms. The results show that, in a static punishment mechanism (SPM), the strategy between governments and enterprises is uncertain. Moreover, the evolutionary trajectory between governments and enterprises is uncertain. However, under the dynamic punishment mechanism (DPM), the evolution path between governments and enterprises tends to converge to a stable value. Thus, the DPM is more conducive than the SPM for industrial pollution control.

## 1. Introduction

In recent years, increasing attention has been paid to environmental pollution. One of the main sources of pollution is industrial pollution, which causes great damage on the environment. Thus, it is important to increase the efficiency of industrial pollution management. Over the past decade, there has been a widespread interest in the field of control theory to discuss environmental pollution [1,2,3,4,5]. However, industrial pollution management is a complex project which needs the participation of local governments and enterprises. Thus, different strategies selected by local governments and enterprises are very important to deal with the industrial pollution. Therefore, this is a decision problem which involves conflicting objectives. As Raquel et al. [6] illustrated, the best way to deal with this class of multi-objective conflict resolution problem is through conflict resolution, which is a special field of game theory. The theory of games was first formalized by Morgenstern and Von Neumann [7] in reference to human economic behavior and has been used in many areas, such as climatic change, emergency, and nuclear accidents. Since then, game theory has developed rapidly. Many scholars have applied this method to environmental pollution management [8,9,10,11,12,13,14,15,16]. For a review of the literature on audit mechanisms using standard game theory, see [8,9,10,15,16]. These audit mechanisms are very important for governments to conduct policy. For example, Cason et al. [8] examined the effectiveness of traditional regulatory schemes and newly emerging social information schemes for achieving compliance. However, the above research was mainly in a static principal-agent framework and based on classical game theory. Luce [17] argued that a central assumption of classical game theory is that players will behave rationally. Such an assumption would clearly be out of place in an evolutionary context.

Friedman and Daniel [18] argued that evolutionary game theory analyses players’ interaction strategy from bounded rationality. In an evolutionary game, each individual chooses among alternative actions or behaviors whose payoff or fitness depends on the choices of others. Thus, evolutionary game theory is a way of thinking about evolution at the phenotypic level when the fitness of particular phenotypes depends on their frequencies in the population [19]. Therefore, evolutionary game theory leads to a new type of ‘solution’ for a game, the ‘evolutionary stable strategy’ (ESS). An ESS is a strategy such that, if all the members of a population adopt it, then no mutant strategy could invade the population under the influence of natural selection.

Based on the assumption of bounded rationality, evolutionary game theory has seized a large and increasing share of game theory literature in recent years. It has been applied to many areas, such as interspecific competition for resources, animal dispersal, land use, plant growth and reproduction [20,21,22,23,24]. For example, Xie [24] provided a case in Hunan province, China, which discussed evolutionary game and the simulation of management strategies of fallow, cultivated land. Additionally, many scholars have applied evolutionary game theory to environmental pollution [25,26,27,28]. For example, Wang et al. [25] built a system dynamics model for studying a mixed-strategy evolutionary game between government and firms. Chen and Hu [26] used evolutionary game theory to study governments’ and manufacturers’ behavioral strategies under various carbon taxes and subsidies. Estalaki et al. [27] applied evolutionary game theory to discuss river water quality management. Shen and Wang [28] established two kinds of government supervision mechanisms based on evolutionary game theory.

To the best of our knowledge, though there is some research about industrial pollution control under the framework of evolutionary games, most of it has been mainly focused on the solution and stability of the equilibrium point. Besides, it has ignored the discussion of the central point. In a real situation, it is difficult for enterprises to strictly reduce emissions. Therefore, research on the central point will be of more practical significance. Our contributions can be summarized as follows: (1) We construct an evolutionary game model for industrial pollution between local governments and enterprises under two punishment mechanisms to study the dynamic evolution path of a game system and the evolutionary stable strategy. Notably, the evolutionary stable strategy that we studied focuses on the stability of the central point. (2) We compare the evolution process of the system under the two mechanisms.

The rest of this paper is organized as follows. Section 2 describes in detail about the evolutionary game model of industrial pollution under the static punishment mechanism (SPM). In Section 3, we describe in detail about the evolutionary game model of industrial pollution under the dynamic punishment mechanism (DPM). Finally, the conclusions of this paper are presented in Section 4.

## 2. The Evolutionary Game Model of Industrial Pollution under the SPM

In this section, an evolutionary game model is presented under the SPM. Furthermore, the evolutionary game model is described in detail as follows.

### 2.1. Research Assumptions

**Assumption** **1.** *There are two main players in the process of industrial pollution management. The first one is the local government, marked as (LG). The second one is the enterprise, marked as (EP). Moreover, we suppose that the two players are finitely rational. In addition, we assume that each player can learn constantly and adjust its strategy to adapt to changes of environment.*

**Assumption** **2.** *In this assumption, we need to define the strategy set of the two players. To illustrate, we assume that there are two strategies for each player. Additionally, we suppose that the strategies contain two opposing evolutionary strategies. Principally, to control the industrial pollution, the local government must take measures to supervise to the enterprise, because the enterprise will discharge of pollutants in the environment. In the process of industrial pollution control, the local government can actively supervise or negatively supervise to the enterprise. To illustrate, we call on the local government to actively supervise, if it shows a strong willingness to supervise. Additionally, we call on the local government to negatively supervise if it shows a weak willingness to supervise. At the same time, the enterprise can also show a strong or weak willingness to reduce emissions.*

**Assumption** **3.** *We suppose that the probability of the local government showing a strong willingness to supervise is p, so the probability of the local government showing a weak willingness to supervise is 1−p. Moreover, the enterprise can also have two choices to reduce emissions. We assume that the probability of the enterprise showing a strong willingness to reduce emissions is q, so the probability of the enterprise showing a weak willingness to reduce emissions is 1−q. It is obvious that p,q∈[0,1], 1−p,1−q∈[0,1].*

### 2.2. Parameters Setting

Due to the fact that the willingness to reduce emissions is different, the enterprise will pay a different cost to reduce emissions. In order to distinguish the cost effectively, we assume that $\xi_{1}$ is the cost of a strong willingness and $\xi_{2}$ is the cost of a weak willingness. The enterprise will pay more when it shows a strong willingness than when it shows weak willingness to reduce emissions. Thus, obviously, $\xi_{1}>\xi_{2}$. Similar to the enterprise, we assume that the cost of the local government with a strong willingness is $\psi_{1}$. If the local government decreases its willingness from strong to weak, the decreased cost is $\psi_{2}$, $\psi_{1}>\psi_{2}$. We suppose that the enterprise will get an extra benefit when it shows a weak willingness to reduce emissions. Then, we assume the extra benefit is e. Moreover, we assume that if the local government shows a weak willingness to supervise, the enterprise will not be punished even if it shows a weak willingness to reduce emissions. On the contrary, we suppose that when the enterprise shows a weak willingness to reduce emissions at the same time as when the local government shows a strong willingness to supervise, the enterprise will be punished by the local government. Furthermore, we assume that the penalty value is fixed, marked as f. In this case, we call the punishment mechanism the SPM. On the contrary, we assume that the penalty value f is dynamic, and the dynamic penalty value is f(p)—we call this punishment mechanism the DPM. The evolutionary game model of industrial pollution under the SPM will be discussed in Section 2. Then, the evolutionary game model of industrial pollution under the DPM will be discussed in Section 3.

Based on the above assumptions, all parameters are shown in Table 1. Additionally, the payoff matrix under the SPM is shown in Table 2.

### 2.3. The Evolutionary Game between the Enterprise and the Local Government

In this section, the evolutionary game model for industrial pollution under the SPM is established. First of all, when the local government shows a strong willingness to supervise, the expected return $E_{p}$ of the enterprise is given by

(1) $$E_{p}=q(-\xi_{1})+(1-q)(-\xi_{1})$$

On the contrary, when the local government shows a weak willingness to supervise, the expected return $E_{1-p}$ of the enterprise is given by

(2) $$E_{1-p}=q(-\xi_{2}+e-f)+(1-q)(-\xi_{2}+e)$$

In addition, we assume that the average benefit ${\bar{E}}_{EP}$ of the enterprise is given by

(3) $${\bar{E}}_{EP}=pE_{p}+(1-p)E_{1-p}=-p\xi_{1}-qf-\xi_{2}+e+pqf+p\xi_{2}-pe$$

Then, according to Equations (1)–(3), the replicated dynamic equation EP(p) of the enterprise can be obtained as follows:

(4) $$\begin{array}{cc} EP(p) & =\frac{dp}{dt}=p(E_{p}-{\bar{E}}_{EP})\;=p(-\xi_{1}+p\xi_{1}+qf+\xi_{2}-e-pqf-p\xi_{2}+pe)\\ & =p(1-p)(-\xi_{1}+qf+\xi_{2}-e) \end{array}$$

Moreover, when the enterprise shows a strong willingness to reduce emissions, we assume that the expected return $E_{q}$ of the government is given by

(5) $$E_{q}=p(-\psi_{1})+(1-p)(-\psi_{1}+f)$$

On the contrary, when the enterprise shows a weak willingness to reduce emissions, we suppose that the expected return $E_{1-q}$ of the government is given by

(6) $$E_{1-q}=p(-\psi_{2})+(1-p)(-\psi_{2})$$

In addition, we assume that the average benefit ${\bar{E}}_{NG}$ of the local government is given by

(7) $${\bar{E}}_{NG}=qE_{q}+(1-q)E_{1-q}=-q\psi_{1}+qf-pqf-\psi_{2}+q\psi_{2}$$

Furthermore, from Equations (5)–(7), we obtain the replicated dynamic equation GN(q) of the local government as follows:

(8) $$\begin{array}{cc} GN(q) & =\frac{dq}{dt}=q(E_{q}-{\bar{E}}_{NG})=q(-\psi_{1}+f-pf+q\psi_{1}-qf+pqf+\psi_{2}-q\psi_{2})\\ & =q(1-q)(-\psi_{1}+f-pf+\psi_{2}) \end{array}$$

Thus, according to Equations (4) and (8), a dynamic system $S_{1}$ is given by

(9) $$\left\{ \begin{array}{l} EP(p)=\frac{dp}{dt}=p(E_{p}-{\bar{E}}_{EP})\;=p(1-p)(-\xi_{1}+qf+\xi_{2}-e)\\ GN(q)=\frac{dq}{dt}=q(E_{q}-{\bar{E}}_{NG})=q(1-q)(-\psi_{1}+f-pf+\psi_{2}) \end{array}\right.$$

#### 2.3.1. The Jacobi Matrix Partial Stability Analysis

According to the method of the Jacobi matrix proposed by Friedman and Daniel [18], we can obtain the evolutionary stability of the replicated dynamic system at the equilibrium point. We suppose that the Jacobi matrix of system $S_{1}$ is J. Thus, we have the corresponding Jacobi matrix as follows:

(10) $$J=\left[\begin{array}{cc} \left(1-2p)(-\xi_{1}+qf+\xi_{2}-e\right) & p(1-p)f\\ -q(1-q)f & \left(1-2q)(-\psi_{1}+f-pf+\psi_{2}\right) \end{array}\right]$$

Then, according to Equation (10), we have:

(11) $$\det J=(1-2p)(-\xi_{1}+qf+\xi_{2}-e)(1-2q)(-\psi_{1}+f-pf+\psi_{2})+p(1-p)f^{2}q(1-q)$$

The trace of the matrix J is:

(12) $$trJ=(1-2p)(-\xi_{1}+qf+\xi_{2}-e)+(1-2q)(-\psi_{1}+f-pf+\psi_{2})$$

According to Equation (9), let EP(p) = 0,GN(q) = 0, so the possible equilibrium points of system $S_{1}$ are: $E_{1}(0,0),E_{2}(0,1),E_{3}(1,0),E_{4}(1,1),E_{5}(p^{*},q^{*})$ among with $p^{*}=\frac{-\psi_{1}+\psi_{2}+f}{f},q^{*}=\frac{\xi_{1}-\xi_{2}+e}{f}$.

Then, from Equations (11) and (12) and the point $E_{5}(p^{*},q^{*})$, we have:

(13) $$\left\{ \begin{array}{cc} \int_{1}:\det J{|}_{\left(p^{*},\;q^{*}\right)} & =(1-2p^{*})(-\xi_{1}+q^{*}f+\xi_{2}-e)(1-2q^{*})(-\psi_{1}+f-p^{*}f+\psi_{2})+p^{*}(1-p^{*})f^{2}q^{*}(1-q^{*})\\ & =\frac{\left(\psi_{2}-\psi_{1}+f)(\psi_{1}-\psi_{2})(\xi_{1}-\xi_{2}+e)(\xi_{2}-\xi_{1}+f-e\right)}{f^{2}}\\ \int_{2}:trJ{|}_{\left(p^{*},\;q^{*}\right)} & =(1-2p^{*})(-\xi_{1}+q^{*}f+\xi_{2}-e)(1-2q^{*})(-\psi_{1}+f-p^{*}f+\psi_{2}\\ & =0 \end{array}\right.$$

Then, the value of the matrix determinant and the trace of the matrix from the five equilibrium points are given in Table 3.

According to evolutionary theory, if the Jacobi matrix satisfies detJ>0,trJ<0, then the corresponding equilibrium point is the locally asymptotically stable fixed point, and the corresponding evolutionary strategy is the evolutionary stability strategy. From Section 2.2, we can see that $\xi_{1}>\xi_{2}$ and $\psi_{1}>\psi_{2}$. Then, we have $\xi_{2}-\xi_{1}-e<0$ and $\psi_{1}-\psi_{2}>0$. Furthermore, the following four cases are discussed.

**Case** **1.** *If $\psi_{2}-\psi_{1}+f>0,\xi_{2}-\xi_{1}+f-e>0$, then the evolutionary stability of local equilibrium points is presented as shown in Table 4.*

**Case** **2.** *If $\psi_{2}-\psi_{1}+f>0,\xi_{2}-\xi_{1}+f-e<0$, then the evolutionary stability of local equilibrium points is presented as shown in Table 5.*

**Case** **3.** *If $\psi_{2}-\psi_{1}+f<0,\xi_{2}-\xi_{1}+f-e>0$, then the evolutionary stability of local equilibrium points is presented as shown in Table 6.*

**Case** **4.** *If $\psi_{2}-\psi_{1}+f<0,\xi_{2}-\xi_{1}+f-e<0$, then the evolutionary stability of local equilibrium points is presented as shown in Table 7.*

**Figure 1:**
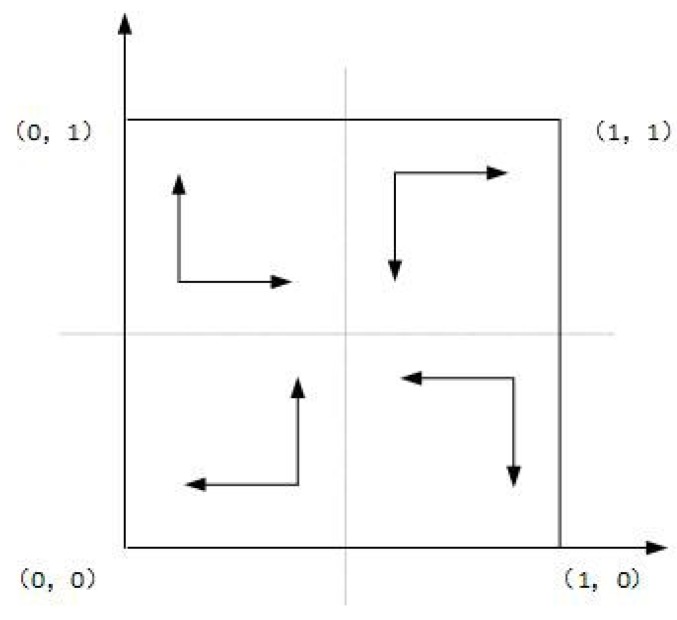
Phase diagram of Case 1.

**Figure 2:**
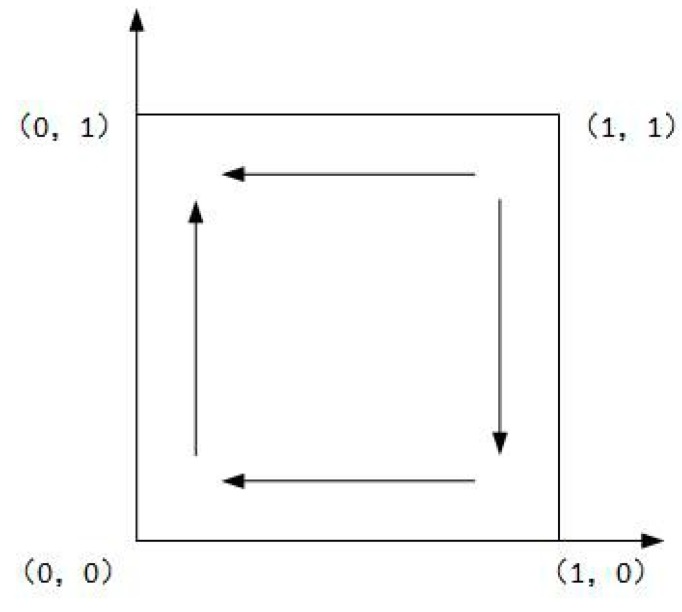
Phase diagram of Case 2.

**Figure 3:**
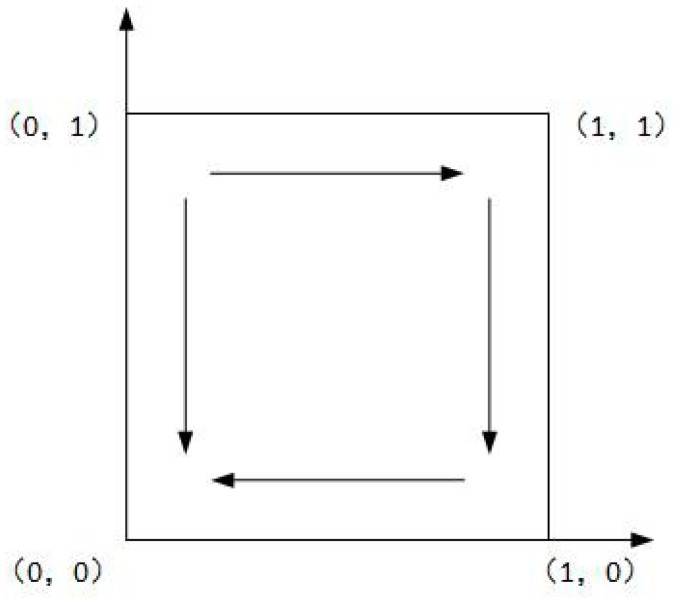
Phase diagram of Case 3.

**Figure 4:**
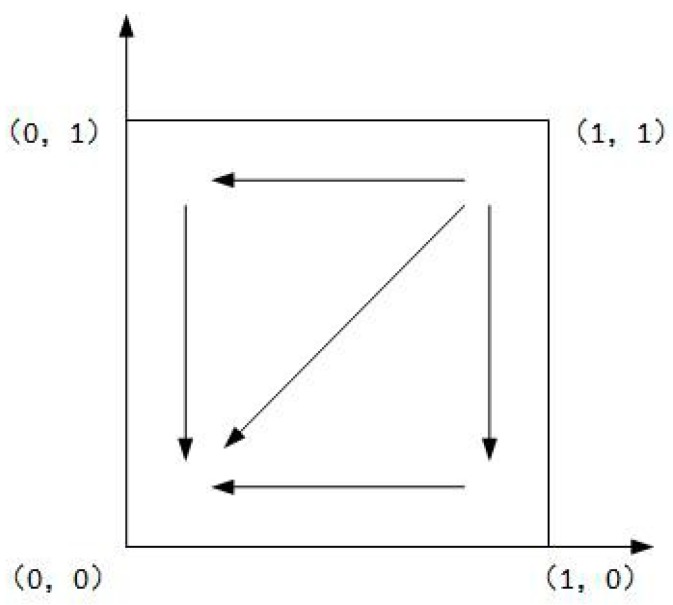
Phase diagram of Case 4.

As shown in Case 1, if $\psi_{2}-\psi_{1}+f>0$, and $\xi_{2}-\xi_{1}+f-e>0$, there are four saddle points and a central point. Then, there is no ESS point. Therefore, in this case, no certain strategy between the local government and the enterprise is reached. As shown in Case 2, if $\psi_{2}-\psi_{1}+f>0$ and $\xi_{2}-\xi_{1}+f-e<0$, then there is a ESS point at (0,1). In this case, the enterprise will completely show a weak willingness to reduce emissions, and the local government will adopt the strategy which shows a strong willingness to supervise. Moreover, as shown in Cases 3 and 4, there is an ESS point at (0,0). Thus, although the enterprise shows a weak willingness to reduce emissions, the local government still shows a weak willingness to supervise. Furthermore, four evolutionary phase diagrams are presented in Figure 1, Figure 2, Figure 3 and Figure 4.

#### 2.3.2. System Simulation Analysis

In order to intuitively observe the dynamic evolution process of the strategy selected between the local government and the enterprise, the MATLAB system simulation tool was used under the four cases. The assumed values of the parameters under the four cases are shown in Table 8.

**Figure 5:**
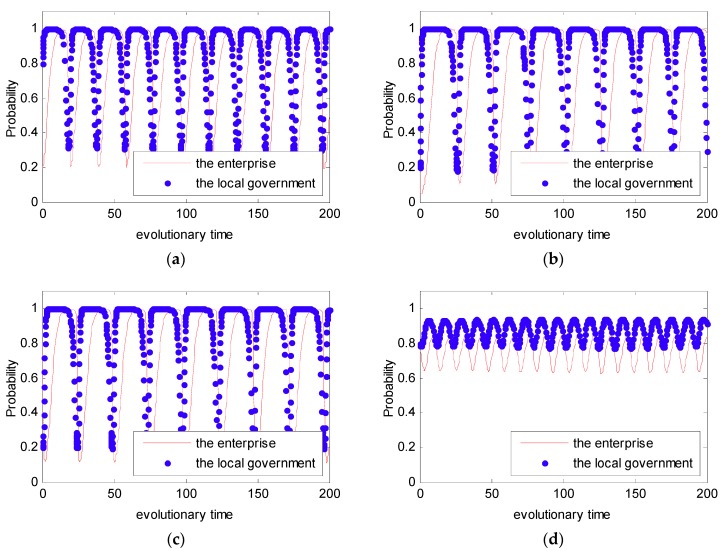
Probability of choosing strong willingness strategy for different time t. (**a**) $p_{t}(0)=0.2,\;q_{t}(0)=0.8$; (**b**) $p_{t}(0)=0.2,\;q_{t}(0)=0.2$; (**c**) $p_{t}(0)=0.8,\;q_{t}(0)=0.2$; and (**d**) $p_{t}(0)=0.8,\;q_{t}(0)=0.8$.

In Case 1, we assumed that the initial value as $\xi_{1}=3,\xi_{2}=1,\psi_{1}=2,\psi_{2}=1,f=4,e=1.5$. Additionally, in order to entirely present the evolutionary game between the local government and the enterprise, four initial states of (p,q) were simulated with different probabilities: (0.2, 0.8), (0.2, 0.2), (0.8, 0.2), (0.8, 0.8). The four initial states stand for four different strategies; that is to say that the strategies selected by the local government and the enterprise were: Weak willingness to reduce emissions, strong willingness to supervise; weak willingness to reduce emissions, weak willingness to supervise; strong willingness to reduce emissions, weak willingness to supervise; and strong willingness to supervise, strong willingness to supervise. The simulation analysis is presented in Figure 5. From Figure 5, we can see that no matter what the initial state is, the strategy between the local government and the enterprise is uncertain. Besides, the strategy between the local government and the enterprise presents periodic concussion. In the following, the evolutionary process between the local government and the enterprise under the DPM is further discussed.

**Figure 6:**
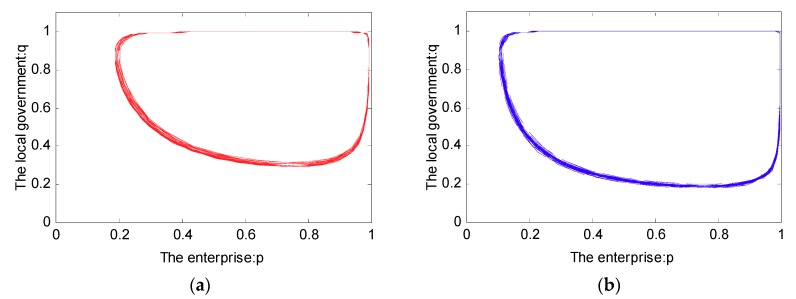
The simulation analysis between the local government and the enterprise. (**a**) $p_{t}(0)=0.2,\;q_{t}(0)=0.8$ and (**b**) $p_{t}(0)=0.8,\;q_{t}(0)=0.2$.

In Case 1, we know that the strategy between the local government and the enterprise is uncertain. Furthermore, let $p_{t}(0)=0.2,q_{t}(0)=0.8$ and $p_{t}(0)=0.8,q_{t}(0)=0.2$; the simulation analysis is presented in Figure 6. As can be seen in Figure 6, the evolutionary path of the behavioral strategy between the local government and the enterprise presents a closed loop. This means that no certain strategies between the local government and the enterprise will be reached in the real world.

**Figure 7:**
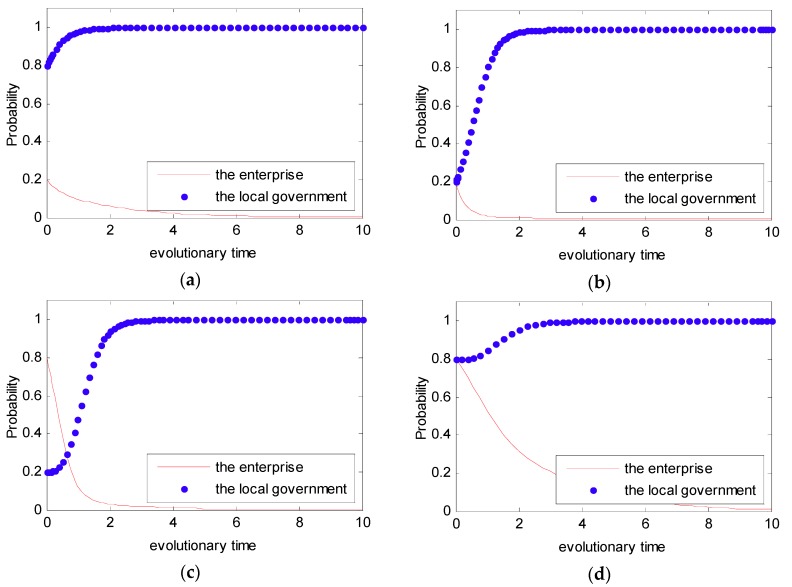
Probability of choosing a strong willingness strategy for different time t. (**a**) $p_{t}(0)=0.2,\;q_{t}(0)=0.8$; (**b**) $p_{t}(0)=0.2,\;q_{t}(0)=0.2$; (**c**) $p_{t}(0)=0.8,\;q_{t}(0)=0.2$; and (**d**) $p_{t}(0)=0.8,\;q_{t}(0)=0.8$.

In Case 2, we assume that the initial value as $\xi_{1}=3,\xi_{2}=1,\psi_{1}=2,\psi_{2}=1,f=4,e=2.5$ to satisfy the condition that $\psi_{2}-\psi_{1}+f>0,\xi_{2}-\xi_{1}+f-e<0$. Additionally, similar to Case 1, four initial states were simulated with different probabilities: (0.2, 0.8), (0.2, 0.2), (0.8, 0.2), (0.8, 0.8). This simulation analysis is presented in Figure 7. From Figure 7, we can see that no matter what the initial state is, the strategy between the local government and the enterprise is certain, and it converges to (0,1). Thus, in this situation, the final evolutional result is that the local government tends to show a strong willingness to supervise, while the enterprise tends to show a weak willingness to reduce emissions.

**Figure 8:**
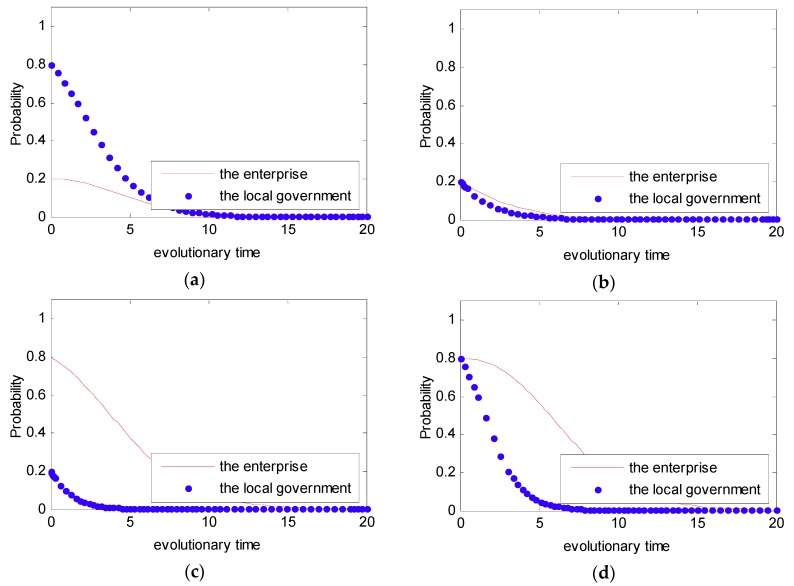
Probability of choosing a strong willingness strategy for different time t. (**a**) $p_{t}(0)=0.2,\;q_{t}(0)=0.8$; (**b**) $p_{t}(0)=0.2,\;q_{t}(0)=0.2$; (**c**) $p_{t}(0)=0.8,\;q_{t}(0)=0.2$; and (**d**) $p_{t}(0)=0.8,\;q_{t}(0)=0.8$.

**Figure 9:**
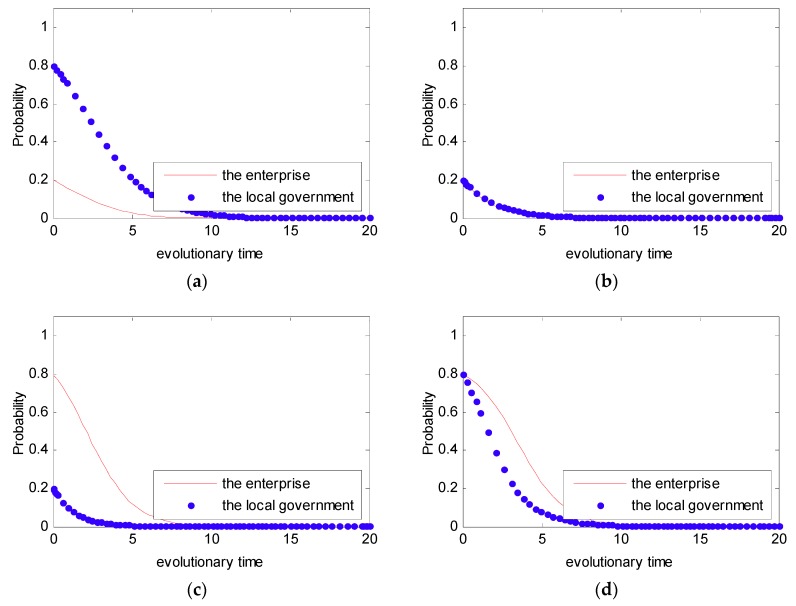
Probability of choosing a strong willingness strategy for different time t. (**a**) $p_{t}(0)=0.2,\;q_{t}(0)=0.8$; (**b**) $p_{t}(0)=0.2,\;q_{t}(0)=0.2$; (**c**) $p_{t}(0)=0.8,\;q_{t}(0)=0.2$; and (**d**) $p_{t}(0)=0.8,\;q_{t}(0)=0.8$.

Similar to Cases 1 and 2, for Cases 3 and 4, four initial states of (p,q) were simulated with different probabilities: (0.2, 0.8), (0.2, 0.2), (0.8, 0.2), (0.8, 0.8). The simulation analyses are presented in Figure 8 and Figure 9. From Figure 8 and Figure 9, we can see that no matter what the initial state is, the strategy between the local government and the enterprise is certain, and it converges to (0,0). Thus, the final evolutional result is that the local government tends to show a weak willingness to supervise, while the enterprise tends to show a weak willingness to reduce emissions.

## 3. The Evolutionary Game Model of Industrial Pollution under the DPM

### 3.1. The Model under the DPM

In this section, we assume that the penalty value f is dynamic, and the dynamic penalty value is f(p). We call this punishment mechanism the DPM. In the DPM, we suppose that the penalty value f is proportional to the probability of the enterprise which shows a weak willingness to reduce emissions. As such, we set $f(p)=(1-p)f_{m}$, where $f_{m}$ is the maximum penalty. Based on the above analysis, a new payoff matrix under the DPM is shown in Table 9.

Based on the payoff matrix under the DPM, we can establish an evolutionary game model. Then, the dynamic system $S_{2}$ combined by the replicated dynamic equation of the local government and the enterprise is given by

(14) $$\left\{ \begin{array}{l} EP(p)=\frac{dp}{dt}=p(E_{p}-{\bar{E}}_{EP})\;=p(1-p)[-\xi_{1}+qf(p)+\xi_{2}-e]\\ GN(q)=\frac{dq}{dt}=q(E_{q}-{\bar{E}}_{NG})=q(1-q)[-\psi_{1}+f(p)-pf(p)+\psi_{2}] \end{array}\right.$$

In the dynamic system (14), let EP(p)=0,GN(q)=0. As such, the possible equilibrium points of system $S_{2}$ are: $E_{1}(0,0),E_{2}(0,1),E_{3}(1,0),E_{4}(1,1),E_{5}(p_{1}{}^{*},q_{1}{}^{*})$ among which $p_{1}{}^{*}=\frac{-\psi_{1}+\psi_{2}+f(p)}{f(p)},q_{1}{}^{*}=\frac{\xi_{1}-\xi_{2}+e}{f(p)}$.

If we suppose that the Jacobi matrix of system $S_{2}$ is $J_{2}$, we can recalculate the corresponding Jacobi matrix as follows:

(15) $$J_{2}=\left[\begin{array}{cc} \left(1-2p)(-\xi_{1}+qf(p)+\xi_{2}-e\right) & p(1-p)f(p)\\ -q(1-q)f(p) & \left(1-2q)(-\psi_{1}+f(p)-pf(p)+\psi_{2}\right) \end{array}\right]$$

According to Section 2.3.1, the strategy selected by the local government and the enterprise is uncertain. In addition, in other cases, the strategy selected by the local government and the enterprise is certain. As such, we just discuss the Case 1 under the DPM.

**Case** **5.** If $\psi_{2}-\psi_{1}+f(p)>0$ and $\xi_{2}-\xi_{1}+f(p)-e>0$. Substituting $E_{5}(p_{1}{}^{*},q_{1}{}^{*})$ into the Equation (14) for the sake of simplicity, we have the evolutionary stability of local equilibrium points in this case as shown in Table 10.

### 3.2. Simulation Analysis

**Figure 10:**
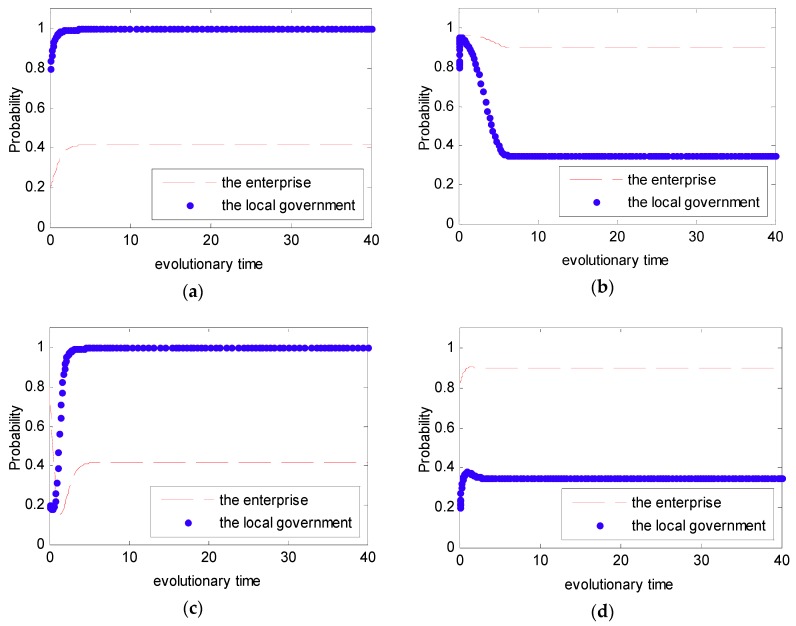
Probability of choosing a strong willingness strategy for different time t. (**a**) $p_{t}(0)=0.2,\;q_{t}(0)=0.8,\;f_{m}=6$; (**b**) $p_{t}(0)=0.2,\;q_{t}(0)=0.8,\;f_{m}=6$; (**c**) $p_{t}(0)=0.8,\;q_{t}(0)=0.2,\;f_{m}=100$; and (**d**) $p_{t}(0)=0.8,\;q_{t}(0)=0.2,\;f_{m}=100$.

In order to intuitively observe the dynamic evolution process of the strategy selected between the government and the enterprise under the DPM, the simulation analysis was used under the Case 1. In Case 1, we set the initial value in the payoff matrix as $\xi_{1}=3,\xi_{2}=1,\psi_{1}=2,\psi_{2}=1,f=4,e=1.5$ to satisfy the condition that $\psi_{2}-\psi_{1}+f>0,\xi_{2}-\xi_{1}+f-e>0$. However, under the DPM, we assume that $f=f(p)=(1-p)f_{m}$, $f_{m}=6$, and $f_{m}=100$. Additionally, in order to present the evolutionary game between the local government and the enterprise, four initial states of $\left(p,q,f_{m}\right)$ were simulated with different probabilities: (0.2, 0.8, 6), (0.2, 0.8, 6), (0.8, 0.2, 100), (0.8, 0.2, 100). The four initial states stand for four different strategies, namely a weak willingness to reduce emissions and a strong willingness to supervise under the condition that $f_{m}=6$ and a strong willingness to reduce emissions and a weak willingness to supervise under the condition that $f_{m}=100$. The simulation analysis is presented in Figure 10.

From Figure 5 and Figure 6, we can see that the strategy between the government and the enterprise is uncertain and presents periodic concussion under the SPM. Moreover, we find that the evolutionary path of the behavioral strategy between the local government and the enterprise presents a closed loop. However, from Figure 10, we can see that no matter what the initial state is, the strategy between the local government and the enterprise is certain. Provided with different probabilities, the probabilities of choosing a strong willingness strategy will converge to different values. Moreover, while the penalty value $f_{m}$ is increased, the probability that the enterprise tends to actively reduce emissions is greatly increased.

## 4. Conclusions

This paper presents an evolutionary game model for industrial pollution under two punishment mechanisms. The strategy between local governments and enterprises is uncertain and presents periodic concussion under the SPM. Moreover, the evolutionary path of the behavioral strategy between local governments and enterprises presents a closed loop. However, under the DPM, the strategy between local governments and enterprises is certain, and it converges to different values. Moreover, while the penalty value $f_{m}$ is increased, the probability that enterprises tend to actively reduce emissions is greatly increased. As such, the results show that the DPM is more conducive than the SPM for industrial pollution control.

From the above results, some recommendations are presented to the policy of local governments. On the one hand, local governments can adopt the DPM when dealing with industrial problems. On the other hand, local governments should actively take some measures to promote enterprises to protect the environment. Moreover, local governments can appeal to the public to participate in environmental supervision to reduce the cost of its supervision.

This paper studied an evolutionary game model for industrial pollution under two punishment mechanisms. However, central governments’ punishments to local governments was not taken into account. As such, a further research direction would be studying situations where central governments’ punishments to local governments are taken into account.

## Figures and Tables

**Table 1:** All parameters.

Parameters	Definitions	Value Range
LG	The local government	-
EP	The enterprise	-
p	Probability of the local government showing a strong willingness to supervise	0≤p≤1
q	Probability of the enterprise showing a strong willingness to reduce emissions	0≤q≤1
$\xi_{1}$	The cost of a strong willingness for the enterprise	$\xi_{1}>0$
$\xi_{2}$	The cost of a weak willingness for the enterprise	$\xi_{2}>0$
$\psi_{1}$	The cost of a strong willingness for the government	$\psi_{1}>0$
$\psi_{2}$	The cost of a weak willingness for the government	$\psi_{2}>0$
e	Extra benefit for the enterprise	e>0
f	The penalty value	f>0

**Table 2:** The payoff matrix under the static punishment mechanism (SPM).

The Enterprise	The Government	
	Strong Willingness (q)	Weak Willingness (1−q)
Strong willingness (p)	$\left(-\xi_{1},-\psi_{1}\right)$	$\left(-\xi_{1},-\psi_{2}\right)$
Weak willingness (1−p)	$\left(-\xi_{2}+e-f,-\psi_{1}+f\right)$	$\left(-\xi_{2}+e,-\psi_{2}\right)$

**Table 3:** All parameters.

Equilibrium Point		Expression
$E_{1}(0,0)$	detJ	$\left(\xi_{2}-\xi_{1}-e)(\psi_{2}-\psi_{1}+f\right)$
	trJ	$\left(\xi_{2}-\xi_{1}-e)+(\psi_{2}-\psi_{1}+f\right)$
$E_{2}(0,1)$	detJ	$\left(\xi_{2}-\xi_{1}-e+f)[-(\psi_{2}-\psi_{1}+f)\right]$
	trJ	$\left(\xi_{2}-\xi_{1}-e+f)-(\psi_{2}-\psi_{1}+f\right)$
$E_{3}(1,0)$	detJ	$\left[-(\xi_{2}-\xi_{1}-e)](\psi_{2}-\psi_{1}\right)$
	trJ	$-(\xi_{2}-\xi_{1}-e)+(\psi_{2}-\psi_{1})$
$E_{4}(1,1)$	detJ	$\left(\xi_{2}-\xi_{1}-e+f)(\psi_{2}-\psi_{1}\right)$
	trJ	$-(\xi_{2}-\xi_{1}-e+f)-(\psi_{2}-\psi_{1})$
$E_{5}(p^{*},q^{*})$	detJ	$\frac{\left(\psi_{2}-\psi_{1}+f)(\psi_{1}-\psi_{2})(\xi_{1}-\xi_{2}+e)(\xi_{2}-\xi_{1}+f-e\right)}{f^{2}}$
	trJ	0

**Table 4:** The evolutionary stability of local equilibrium points in Case 1.

Equilibrium Point	detJ	trJ	Results
$E_{1}(0,0)$	−	±	Saddle point
$E_{2}(0,1)$	−	±	Saddle point
$E_{3}(1,0)$	−	±	Saddle point
$E_{4}(1,1)$	−	±	Saddle point
$E_{5}(p^{*},q^{*})$	+	±	Central point

**Table 5:** The evolutionary stability of local equilibrium points in Case 2.

Equilibrium Point	detJ	trJ	Results
$E_{1}(0,0)$	−	±	Saddle point
$E_{2}(0,1)$	+	−	ESS
$E_{3}(1,0)$	−	±	Saddle point
$E_{4}(1,1)$	±	+	Unstable point
$E_{5}(p^{*},q^{*})$	−	0	Central point

**Table 6:** The evolutionary stability of local equilibrium points in Case 3.

Equilibrium Point	detJ	trJ	Results
$E_{1}(0,0)$	+	−	ESS
$E_{2}(0,1)$	+	+	Unstable point
$E_{3}(1,0)$	−	±	Saddle point
$E_{4}(1,1)$	−	±	Saddle point
$E_{5}(p^{*},q^{*})$	−	0	Central point

**Table 7:** The evolutionary stability of local equilibrium points in Case 4.

Equilibrium Point	detJ	trJ	Results
$E_{1}(0,0)$	+	−	ESS
$E_{2}(0,1)$	−	±	Saddle point
$E_{3}(1,0)$	−	±	Saddle point
$E_{4}(1,1)$	+	+	Unstable point
$E_{5}(p^{*},q^{*})$	+	0	Central point

**Table 8:** The assumed values of the parameters under the four cases.

Parameters	Case 1	Case 2	Case 3	Case 4
$\xi_{1}$	3	3	1.2	1.2
$\xi_{2}$	1	1	1	1
$\psi_{1}$	2	2	2	2
$\psi_{2}$	1	1	1	1
f	4	4	0.5	0.5
e	1.5	2.5	0.2	0.5

**Table 9:** The payoff matrix under the dynamic punishment mechanism (DPM).

The Enterprise	The Government	
	Strong Willingness (q)	Weak Willingness (1−q)
Strong willingness (p)	$\left(-\xi_{1},-\psi_{1}\right)$	$\left(-\xi_{1},-\psi_{2}\right)$
Weak willingness (1−p)	$\left(-\xi_{2}+e-f(p),-\psi_{1}+f(p)\right)$	$\left(-\xi_{2}+e,-\psi_{2}\right)$

**Table 10:** The equilibrium points in Case 1 under the DPM.

Equilibrium Point	detJ	trJ	Results
$E_{1}(0,0)$	−	±	Saddle point
$E_{2}(0,1)$	−	±	Saddle point
$E_{3}(1,0)$	−	±	Saddle point
$E_{4}(1,1)$	−	±	Saddle point
$E_{5}(p^{*},q^{*})$	+	0	Central point
